# Patient preference attributes in eHealth interventions for cancer‐related fatigue: A scoping review

**DOI:** 10.1111/ecc.13754

**Published:** 2022-11-16

**Authors:** Lian Beenhakker, Annemieke Witteveen, Kim A. E. Wijlens, Ester J. M. Siemerink, Marije L. van der Lee, Christina Bode, Sabine Siesling, Miriam M. R. Vollenbroek‐Hutten

**Affiliations:** ^1^ Department of Biomedical Signals and Systems University of Twente Enschede The Netherlands; ^2^ Department of Internal Medicine Ziekenhuis Groep Twente Hengelo The Netherlands; ^3^ Scientific Research Department Helen Dowling Institute Bilthoven The Netherlands; ^4^ Department of Medical and Clinical Psychology, Center of Research on Psychology in Somatic diseases Tilburg University Tilburg The Netherlands; ^5^ Department of Psychology, Health and Technology University of Twente Enschede The Netherlands; ^6^ Department of Health Technology and Services Research, Technical Medical Centre University of Twente Enschede The Netherlands; ^7^ Department of Research and Development Netherlands Comprehensive Cancer Organisation (IKNL) Utrecht The Netherlands; ^8^ Board of Directors Medisch Spectrum Twente Enschede The Netherlands

**Keywords:** breast cancer, cancer‐related fatigue, eHealth, interventions, patient preference, scoping review

## Abstract

**Introduction:**

Cancer‐related fatigue (CRF) is one of the most reported long‐term effects breast cancer patients experience after diagnosis. Many interventions for CRF are effective, however, not for every individual. Therefore, intervention advice should be adjusted to patients' preferences and characteristics. Our aim was to develop an overview of eHealth interventions and their (preference sensitive) attributes.

**Methods:**

eHealth interventions were identified using a scoping review approach. Eligible studies included breast cancer patients and assessed CRF as outcome. Interventions were categorised as physical activity, mind–body, psychological, ‘other’ or ‘combination’. Information was extracted on various (preference sensitive) attributes, like duration, intensity, peer support and costs.

**Results:**

Thirty‐five interventions were included and divided over the intervention categories. (Preference sensitive) attributes varied both within and between these categories. Duration varied from 4 weeks to 6 months, intensity from daily to own pace. Peer support was present in seven interventions and costs were known for six.

**Conclusion:**

eHealth interventions exist in various categories, additionally, there is much variation in (preference sensitive) attributes. This provides opportunities to implement our overview for personalised treatment recommendations for breast cancer patients struggling with CRF. Taking into account patients' preferences and characteristics suits the complexity of CRF and heterogeneity of patients.

## 1 INTRODUCTION

In the Netherlands, one in seven women is diagnosed with breast cancer at some point in their lives (Eijkelboom et al., 2020). Early diagnosis, for example by the national screening programme, and improved treatment have increased the survival rates over the years (Netherlands Cancer Registry, 2021). As the number of breast cancer survivors increases, there are more survivors suffering from the long‐term effects of having had cancer and its treatment. One of the most prevalent, but still underreported, long‐term effects is cancer‐related fatigue (CRF) (Bower et al., 2006; de Ligt et al., 2019; Minton & Stone, 2008; Ruiz‐Casado et al., 2021). The National Comprehensive Cancer Network (NCCN) defines CRF as ‘a distressing, persistent, subjective sense of physical, emotional, and/or cognitive tiredness or exhaustion related to cancer or cancer treatment that is not proportional to recent activity and interferes with usual functioning’ (Berger et al., 2020).

Many different interventions exist to help patients in their struggles to prevent or cope with CRF. The NCCN guidelines (Berger et al., 2020) describe two broad types of interventions, pharmacological and non‐pharmacological. The latter is divided into different categories of which physical activity, mind–body therapy and psychological interventions are the most prevalent (Berger et al., 2020; Pearson et al., 2018). In physical activity, patients are motivated to exercise to increase energy expenditure (Conn et al., 2011; Pearson et al., 2018), examples of mind–body interventions are mindfulness, meditation or yoga (Carlson et al., 2017; Pearson et al., 2018), and psychological interventions consists of, for example, psychoeducation, cognitive behavioural therapy (CBT) or supportive‐expressive therapy (Fors et al., 2010; Pearson et al., 2018). Instead of focussing on one specific category, it is also possible to combine categories within one intervention (Pearson et al., 2018).

Non‐pharmacological interventions are often delivered in a face‐to‐face setting. However, patients experience several barriers to follow face‐to‐face interventions, amongst others travel time, costs and lack of transportation (Stubblefield, 2017). To overcome these barriers, interventions can also be delivered online, as eHealth interventions. There is not one clear definition for eHealth, but health and technology are two common terms (Oh et al., 2005). Therefore, in this paper, we define eHealth interventions as *health* interventions that have an online, or *technological*, component that is necessary and relevant to support patients throughout the intervention, with or without healthcare professional. These can be mobile phone applications, websites with assignments or blended care with additional face‐to‐face consults, but not solely a conferencing system to facilitate tele‐consults without additional intervention components.

Reviews report varying results on the effectiveness of interventions on CRF. This holds for both face‐to‐face interventions and eHealth interventions (Corbett et al., 2019; Jiang et al., 2020; Mustian et al., 2017; Myrhaug et al., 2020; Seiler et al., 2017; Van Vulpen et al., 2020; Vannorsdall et al., 2020; Xu et al., 2019). Meta‐analyses showed that interventions can improve fatigue, although not all studies showed this effectiveness (Mustian et al., 2017; Myrhaug et al., 2020; Seiler et al., 2017; Van Vulpen et al., 2020; Vannorsdall et al., 2020; Xu et al., 2019).

Effectiveness of an intervention on an outcome measure, for example CRF, is important to decide what intervention an individual patient should follow. Hilfiker et al. (2018) ranked categories of interventions by effectiveness on CRF to help patients and healthcare professionals decide what intervention to follow. However, even though an intervention is found to be effective in general, it might not help an individual patient. Randomised controlled trials (RCTs) reporting an overall significant effect on CRF also revealed that not all patients showed clinically relevant change; some patients even worsened (Abrahams et al., 2017; Bruggeman‐Everts et al., 2017; Yun et al., 2012). Furthermore, there is not one gold‐standard intervention that works best for all patients with CRF (Bower, 2014). So, as Hilfiker et al. (2018) also suggested, it is relevant to look into preferences of patients when suggesting an intervention.

Factors like personal characteristics and preferences influence whether a patient follows an intervention as intended. For example, participation in RCTs is lower compared to participation in randomised patient preference trials, where patients are divided to their preferred arm (Wasmann et al., 2019). Another example relates to the categories of interventions, a patient who exercised before diagnosis is more likely to successfully follow an exercise intervention (Pickett et al., 2002). In contrast, if an intervention does not fit the personal characteristics and preferences of an individual patient, this might cause a drop in motivation, less time investment and thus fewer to no impact on the outcome measure (Cillessen et al., 2020). Therefore, to do justice to the individual, when advising an intervention, this advice should be personalised to the individual patient, combining the type and severity of CRF, personal characteristics and preferences regarding interventions.

In case preferences of patients can be related to attributes of interventions, these attributes can help patients and healthcare professionals by selecting an intervention that matches patients most, or at least have as many overlap with preferences as possible. For example, flexibility regarding duration and intensity can be an important attribute since patients have different time investment possibilities. Additional preference sensitive attributes in eHealth are having an introductory training, contact with a healthcare professional (HCP), peer support, mode of content delivery, costs and effectiveness (Phillips et al., 2021).

To be able to link patient preferences to attributes of existing interventions, an overview is needed in which these two aspects are combined. Within the best of our knowledge, attributes of interventions related to patient preferences have not been reviewed in combination with existing eHealth interventions. This study therefore aims to answer two research questions: (1) What eHealth interventions exist to help breast cancer patients with CRF? and (2) What (preference sensitive) attributes make up these interventions?

## 2 METHODS

To create an overview of eHealth interventions for CRF in breast cancer patients, a scoping review approach was used, as this fits the broad scope of this study (Arksey & O'Malley, 2005; Tricco et al., 2018). For reporting, the Preferred Reporting Items for Systematics Reviews‐Scoping Review (PRISMA‐ScR) checklist (Tricco et al., 2018) was used where possible.

### 2.1 Search strategy

Up to 3 May 2021, we searched systematically through Scopus (title/abstract/keywords), PubMed (title/abstract) and Web of Science (topic), which includes Medline. To find eligible studies, we used the terms [cancer] AND [intervention*] AND [fatigue OR ‘cancer‐related fatigue’ OR ‘cancer related fatigue’ OR CRF] AND [eHealth OR mHealth OR web‐based OR smartphone OR mobile OR internet‐deliver* OR telehealth OR online]. After abstract and full‐text screening (see below), additional searches were performed for those studies of which (1) only a protocol was included, (2) no information was reported on patient characteristics in relation to adherence and drop‐out or (3) ‘information was reported elsewhere’. This was done by looking at the references and ‘cited by’ list, by finding more papers from the authors and by searching for the name of the intervention (up to 11 June 2021).

### 2.2 Eligibility criteria

We aimed to find eHealth interventions in which (1) the study population included or completely consisted of breast cancer patients and (2) CRF was assessed as (one of the) outcome measure(s). Interventions could be studied with any design and were excluded if they only described the development. The specific eligibility criteria are outlined in Table 1.

**TABLE 1:** Inclusion and exclusion criteria to select the eligible studies in this scoping review

Inclusion criteria	Exclusion criteria
Breast cancer patients were part of the included patient group. All included patients were adults (≥18 years). The studied intervention is an eHealth intervention (as defined in introduction). CRF experienced by patients was assessed before and after the intervention, to determine the effectiveness on CRF, not necessarily as primary outcome. The paper was published up to 3 May 2021 and written in English.	Paediatric cancer survivors were part of the included patient group. The studied intervention was a pharmacological intervention. Only the development of the intervention was described, without any preliminary results or protocol for further research. The paper was a review, letter to the editor, case study or had only an abstract available

### 2.3 Data extraction and synthesis

The papers found with the systematic search were downloaded and combined in Microsoft Excel versions 2103–2205 (through automatic updates). L. B. first screened all titles and removed those duplicate and ineligible. Then, both A. W. and L. B. screened the abstracts and, in case of disagreement, discussed what abstracts to include. The full texts were downloaded into Mendeley version 1.19.8. L. B. read all full texts and discussed with S. S., M. V., K. W. and A. W. to reach consensus on which to include. If interventions were described in several papers, information was combined.

For all interventions, information was extracted and charted into an overview in Excel. The information was based on several data items (see Table 2), which were related to general information of the intervention, (preference sensitive) attributes and patient characteristics. The included attributes are those mentioned in the introduction, extended with attributes that followed from the consultation sessions (see below). Information on HCP contact included if the contact was real‐time (synchronous) or via, for example, email (asynchronous).

**TABLE 2:** Overview of data items extracted to compare the different interventions

General information on interventions
Author/year of publication
Name intervention
Explanation intervention
Follow‐up period after intervention
(Description of) category of intervention + references why chosen for this category
CRF assessed as primary or secondary outcome
Questionnaire to assess CRF
Type of study
Total number of participants and % breast cancer patients
Language/country of intervention
Recruitment/how to find intervention
Inclusion and exclusion criteria, extra focus on time since primary treatment and age

(Preference sensitive) attributes and patient characteristics
Duration
Intensity
Usage by participants
Introduction training
HCP contact, including possible contact with the research team during the study
Peer support
Mode of content delivery
Costs of intervention
Effectiveness of intervention (also at follow‐up if studied)
Experiences with intervention
Patient characteristics of successful, adherent and dropped out participants
Reasons for drop‐out

To compare the eHealth interventions, interventions were divided into the three non‐pharmacological categories described in the introduction (physical activity, mind–body and psychological). In case neither or more than one of these three categories fit, interventions were placed in the ‘other’ or ‘combination’ category. The (preference sensitive) attributes and patient characteristics related to successful, adherent and dropped‐out patients were analysed one by one to compare them, if possible, between the intervention categories.

### 2.4 Consultation

As an addition to the PRISMA‐ScR checklist, the methodological framework of Arksey and O'Malley (2005) proposes to add consultation. Therefore, we held two meetings for consultation: one with experts on CRF and one with experts on the user perspective (November 2021). In these meetings, preliminary results were presented, and experts were asked to give input on these results. The input was processed to get to the results presented below.

## 3 RESULTS

**FIGURE 1:**
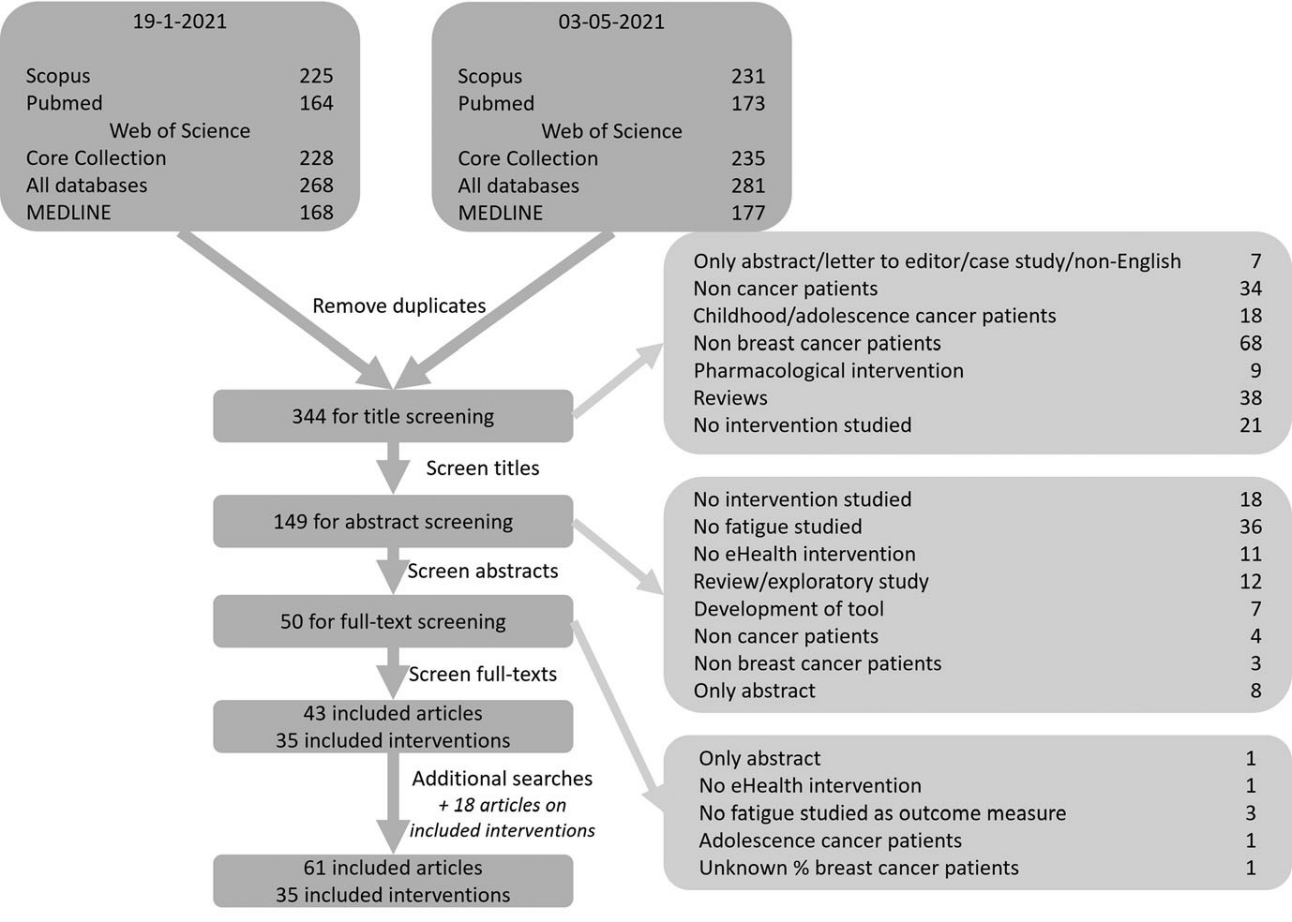
Flow diagram of selection of eligible studies

We identified 344 unique articles of which 43 matched the eligibility criteria. These articles described a total of 35 interventions. With additional searches for more information on the interventions, an extra 18 articles were identified and included as well. Figure 1 shows the flow diagram of the selection of studies. Table S1 shows the full overview of all interventions with all information related to the data items.

### 3.1 eHealth interventions

To answer the first research question, ‘what eHealth interventions exist to help breast cancer patients with CRF?’, the 35 interventions are described below.

For most interventions, the study was performed in the United States (*n* = 12) (Cairo et al., 2020; Henry et al., 2018; Kapoor & Nambisan, 2020; Kelleher et al., 2021; Kubo et al., 2018; Lengacher et al., 2018; Nápoles et al., 2019; Owen et al., 2017; Price‐Blackshear et al., 2020; Ritterband et al., 2012; Smith et al., 2019; Spahrkäs et al., 2020b), followed by the Netherlands (*n* = 6) (Abrahams et al., 2015; Dozeman et al., 2017; van den Berg et al., 2012; Willems et al., 2015; Wolvers et al., 2015). A few interventions (*n* = 3) (Ritterband et al., 2012; Spahrkäs et al., 2020b; Urech et al., 2018; Zachariae et al., 2018) were studied in multiple countries. For more than half of the interventions (*n* = 18), the study was performed only in breast cancer patients (Abrahams et al., 2015; Cairo et al., 2020; Delrieu, Anota, et al., 2020; Dozeman et al., 2017; Galiano‐Castillo et al., 2013; Henry et al., 2018; Holtdirk et al., 2020; Kapoor & Nambisan, 2020; Kelleher et al., 2021; Lee et al., 2014; Lengacher et al., 2018; Mendes‐Santos et al., 2019; Nápoles et al., 2019; Price‐Blackshear et al., 2020; Smith et al., 2019; van den Berg et al., 2012; Zachariae et al., 2018; Zhou et al., 2020), and in studies with mixed samples (*n* = 14), a mean of 52% of the participants had breast cancer (Bray et al., 2017; Bruggeman‐Everts et al., 2017; Falz et al., 2021; Foster et al., 2016; Kubo et al., 2018; Mikolasek et al., 2021; Owen et al., 2017; Puszkiewicz et al., 2016; Spahrkäs et al., 2020b; Urech et al., 2018; Willems, Bolman, et al., 2017; Yun et al., 2012; Zernicke et al., 2016). For three interventions, no percentage breast cancer patients was known yet, as only a protocol was available (Carlson et al., 2019; Corbett et al., 2016; Subnis et al., 2020). In total, we found seven interventions for which only a protocol was available (Carlson et al., 2019; Corbett et al., 2016; Falz et al., 2021; Kapoor & Nambisan, 2020; Kelleher et al., 2021; Mendes‐Santos et al., 2019; Subnis et al., 2020). Ten interventions reported long‐term results, varying from 2 to 12 months after finishing the intervention (Bray et al., 2017; Bruggeman‐Everts et al., 2017; Foster et al., 2016; Galiano‐Castillo et al., 2016; Holtdirk et al., 2021; Urech et al., 2018; van den Berg et al., 2015; Willems, Mesters, et al., 2017; Wolvers, 2017; Zachariae et al., 2018). Details on these results can be found in Table S1. eHealth interventions are relatively new, the oldest articles were published in 2012 (Ritterband et al., 2012; van den Berg et al., 2012; Yun et al., 2012). Also, more than half of the studies (n = 21) were published in the last five years (2017–2021) (Bray et al., 2017; Cairo et al., 2020; Carlson et al., 2019; Delrieu et al., 2018; Dozeman et al., 2017; Falz et al., 2021; Henry et al., 2018; Holtdirk et al., 2020; Kapoor & Nambisan, 2020; Kelleher et al., 2021; Kubo et al., 2018; Lengacher et al., 2018; Mendes‐Santos et al., 2019; Mikolasek et al., 2018; Nápoles et al., 2019; Owen et al., 2017; Price‐Blackshear et al., 2020; Smith et al., 2019; Spahrkäs et al., 2020a; Subnis et al., 2020; Zhou et al., 2020).

Fatigue was assessed using several questionnaires, the Brief Fatigue Inventory (BFI) was used most often (*n* = 7) (Grimmett et al., 2013; Holtdirk et al., 2020; Kapoor & Nambisan, 2020; Kubo et al., 2018; Lee et al., 2014; Mendes‐Santos et al., 2019; Yun et al., 2012). The Patient‐Reported Outcomes Measurement Information System (PROMIS, Henry et al., 2018; Kelleher et al., 2021; Mikolasek et al., 2018; Nápoles et al., 2019; Price‐Blackshear et al., 2020; Subnis et al., 2020) and the Functional Assessment of Cancer Therapy (FACT) or Functional Assessment of Chronic Illness Therapy (FACIT, Bray et al., 2017; Carlson et al., 2019; Grossert et al., 2016; Puszkiewicz et al., 2016; Smith et al., 2019; Zachariae et al., 2018) was used in six interventions. FACT and FACIT are the same as the questionnaire started as FACT and continued later as FACIT (Webster et al., 2003).

We found five (14%) physical activity interventions, which included exercises and activity goal setting (Table 3, Bruggeman‐Everts et al., 2017; Delrieu et al., 2018; Delrieu, Anota, et al., 2020; Delrieu, Pialoux, et al., 2020; Falz et al., 2021; Galiano‐Castillo et al., 2013, 2017, 2016; Puszkiewicz et al., 2016; Wolvers, 2017; Wolvers et al., 2015; Wolvers & Vollenbroek‐Hutten, 2015). The seven (20%) mind–body interventions were almost all mindfulness‐based and are outlined in Table 4 (Carlson et al., 2019; Kubo et al., 2018; Lengacher et al., 2018; Mikolasek et al., 2018, 2021; Price‐Blackshear et al., 2020; Subnis et al., 2020; Zernicke et al., 2014, 2013, 2016). Table 5 shows the psychological interventions (*n* = 13, 37%), which included amongst others CBT, coping skills training and self‐management (Abrahams et al., 2017, 2015; Bray et al., 2017; Corbett et al., 2016; Dozeman et al., 2017; Foster et al., 2015, 2016; Grimmett et al., 2013; Henry et al., 2018; Kanera et al., 2017; Kanera, Bolman, et al., 2016; Kanera, Willems, et al., 2016; Kelleher et al., 2021; Mendes‐Santos et al., 2019; Myall et al., 2015; Owen et al., 2017; Ritterband et al., 2012; van den Berg et al., 2013; van den Berg et al., 2015, 2012; Willems et al., 2015; Willems, Bolman, et al., 2017; Willems, Lechner, et al., 2017; Willems, Mesters, et al., 2017; Yun et al., 2012; Zachariae et al., 2018). Two (6%) interventions did not fit in either of these categories, namely, support with the survivorship care plan and health and wellness coaching (Table 6, Cairo et al., 2020; Kapoor & Nambisan, 2020). Lastly, Table 7 shows eight (23%) interventions with a combination of categories, most combined the mind–body and psychological categories (Bruggeman‐Everts et al., 2015; Bruggeman‐Everts et al., 2017; Grossert et al., 2016; Holtdirk et al., 2021, 2020; Lee et al., 2014; Nápoles et al., 2019; Smith et al., 2019; Spahrkäs et al., 2020a, 2020b; Urech et al., 2018; Wolvers, 2017; Wolvers et al., 2015; Zhou et al., 2020).

**TABLE 3:** Overview of the physical activity interventions and their aspects. In bold at the top, the range of the patient preference sensitive attributes is summarised

Author/year	Name	Type of intervention	Duration	Intensity	Intensity of usage	HCP contact	(A)synchronous contact	Type of study	#participants [started (finished)]	Relation to treatment	Primary/secondary outcome	Questionnaire	Study results
		Range of patient preference sensitive items	6 weeks–6 months	3 sessions/week–3 h/weeks—tailored by users		4/5 with HCP contact	Most have synchronous contact			1× during—4× after			2/5 with significant improvement
(Delrieu et al., 2018; Delrieu, Anota, et al., 2020; Delrieu, Pialoux, et al., 2020)	ABLE	Reach step goal of 10,000 steps/week and 150 min of moderate to vigorous physical activity.	6 months	At least three walking session of at least 10 min per week.	96% of patients wore activity tracking for more than one consecutive week, 54% accumulated more than 5,000 steps per day (sedentary threshold)	Physical activity instructor calls to adjust step goals	Synchronous contact	Single‐arm feasibility trial	51 (49)	During chemotherapy	Secondary	EORTC‐QLQ‐C30—fatigue and PFS‐R	EORTC: decreased by 16% (*p* = 0.07); PFS‐R: no difference (*p* > 0.99)
	ABLE02							Protocol for RCT	Goal: 244		Primary	EORTC‐QLQ‐C30 – Fatigue	Ongoing
(Falz et al., 2021)	CRBP‐TS	Exercises to improve oxygen capacity.	6 months	2–3 sessions per week, 30 min/session	—	Interdisciplinary team supervises the programme	Synchronous contact	Protocol for RCT	Goal: 300	After treatment, max. 6 months	Secondary	EORTC‐QLQ‐C30—fatigue and FSS	Ongoing
(Galiano‐Castillo et al., 2013, 2017, 2016)	e‐CUIDATE	Range of exercises for aerobic and resistance training.	8 weeks	3 × 90 min/week	22.5 ± 1.7 of 24 scheduled sessions were completed	The research team sets up weekly goals and gives feedback to exercises. Possibility for meetings via conferencing system	Synchronous contact	RCT	81 (76)	After treatment	Secondary	PFS‐R and BFS	ES: d = −0.89, 95% CI [−1.30, −0.48]*
(Puszkiewicz et al., 2016)	GAINFitness	Range of exercises: cardiovascular fitness, strength training, yoga, and Pilates exercises	6 weeks	Tailored by user through app	Usage of 2.1 ± 0.7 times per week, each session lasted 25.1 ± 8.2 min	No	—	One‐arm pre‐post design	11 (11)	After treatment	—	FACIT‐F	*z* = −1.27 (*p* = 0.242)
(Bruggeman‐Everts et al., 2017; Wolvers, 2017; Wolvers et al., 2015; Wolvers & Vollenbroek‐Hutten, 2015)	More fit after cancer [*Fitter na kanker*]—AAF	Physical activity was stimulated by showing an activity goal with reference line	9 weeks	3 h/week	—	Weekly feedback is provided	Asynchronous contact	Three‐armed RCT	167 (139)	After treatment, min. 3 months	Primary	CIS	Chi^2^(2) = 28.28 (*p* < 0.001). ES: 1.18*

Abbreviations: BFS, Borg Fatigue Scale; CI, confidence interval; CIS, Checklist for Individual Strength; EORTC‐QLQ‐C30, European Organization for Research and Treatment Cancer Quality of Life Questionnaire—general; FACIT‐F, Functional Assessment of Chronic Illness Therapy—Fatigue; FSS, Fatigue Severity Scale; HCP contact, contact with healthcare professional; PFS‐R, Piper Fatigue Scale—Revised; RCT, randomised controlled trial.

* Significant improvement.

**TABLE 4:** Overview of the mind–body interventions and their aspects. In bold at the top, the range of the patient preference sensitive attributes is summarised

Author/year	Name	Type of intervention	Duration	Intensity	Intensity of usage	HCP contact	(A)synchronous contact	Type of study	#participants [started (finished)]	Relation to treatment	Primary/secondary outcome	Questionnaire	Study results
		Range of patient preference sensitive items	4–12 weeks, outlier of 20 weeks	Daily practice of exercises		2/7 with HCP contact	Both have synchronous contact			2× during—4× after—1× both			4/7 with significant improvement
(Subnis et al., 2020)	Am mindfulness—AmDTx	MBCR	4 weeks	20–30 min/day, min. 4 days/week	—	No	—	Protocol for RCT	Needed: 54; goal: 74	After treatment, min. 2 weeks	Secondary	PROMIS—Cancer bank v1.0—fatigue	Ongoing
(Price‐Blackshear et al., 2020)	C‐MBI/I‐MBI	MBRE and MBSR	8 weeks	1 h/week video and at own pace 10/20/30 min guided meditations.	Self‐reported: 77% watched all sessions, 90% used supplemental meditations, 91% completed some to all homework assignments	No	—	RCT (Phase I)	117 (73)	After treatment, 1–6 years post‐diagnosis	Secondary	PROMIS 29—fatigue domain	*F*(1,148) = 17.56 (*p* < 0.001)*
(Zernicke et al., 2014, 2013, 2016)	eCALM	MBCR	8 weeks	Daily home practice (45 min) weekly 2‐h sessions, online 6‐h weekend retreat	6 ± 3 of 9 classes were attended, home mediation was done for 134 min per week	Instructor conducted weekly 2‐h sessions	Synchronous	RCT	62 (57)	After treatment, max. 3 years	—	POMS—Fatigue	ES: 0.44, *F*(1,113) = 3.95 (*p* = 0.049)*
								Pre‐post analysis of both RCT groups	62 (51)				*F*(1,48.24) = 23.97 (*p* < 0.001)*
(Kubo et al., 2018)	Headspace	Mindfulness	8 weeks	10–20 min/day	71% practiced meditation for >50% of the days. After intervention, 64% mediated at least once.	No	—	Pilot feasibility study	28 (19)	During treatment	—	BFI	Change −0.3 ± 0.8 (*p* > 0.05)
(Mikolasek et al., 2018, 2021)	Mindfulness and relaxation app	Mind–body medicine	20 weeks	15 min/exercise, at own pace, but daily use (5 days/week) advised).	25% used app continuously (once per week). Median exercises completed, for all users: 2 in week 1, 0 in week 9; for continuous users: 6 in week 1, 3–5 in other weeks	No	—	Feasibility study, mixed methods approach	100 (72)	Both during and after treatment	—	PROMIS 29—fatigue domain	ES = −0.38, 95% CI [−0.69, −0.07]. Significant decrease in fatigue over time (*p* = 0.01)*
(Lengacher et al., 2018)	mMBSR (BC)	MBSR	6 weeks	Practice: 15–45 min/days session: 2 h/week	Average of 36 min/day	No	—	Single‐group pre‐post test design	15 (13)	After treatment, 2 weeks–2 years	—	FSI	ES fatigue symptom = 0.60, 95% CI [−0.16, 1.35] (*p* = 0.002). ES fatigue interference = 0.47, 95% CI [−0.28, 1.22] (*p* = 0.03)*
(Carlson et al., 2019)	ONE‐MIND	MBCR	12 weeks	Practice: 30–45 min/day, real‐time session: 55 min/week.	—	Instructor guided weekly 55‐min session	Synchronous	Protocol for RCT	Goal: 178	During treatment	Primary	FACIT‐F	Ongoing

Abbreviations: BFI, Brief Fatigue Inventory; CI, confidence interval; FACIT‐F, Functional Assessment of Chronic Illness Therapy—Fatigue; FSI: Fatigue Symptom Inventory; HCP contact, contact with healthcare professional; MBCR, Mindfulness‐based Cancer Recovery; MBRE, Mindfulness‐based Relationship Enhancement; MBSR, Mindfulness‐based Stress Reduction; POMS, profile of mood states; PROMIS, Patient‐Reported Outcomes Measurement Information System; RCT, randomised controlled trial.

* Significant improvement.

**TABLE 5:** Overview of the found psychological interventions and their aspects. In bold at the top, the range of the patient preference sensitive attributes is summarised

Author/year	Name	Type of intervention	Duration	Intensity	Intensity of usage	HCP contact	(A)synchronous	Type of study	#participants [started (finished)]	Relation to treatment	Primary/secondary outcome	Questionnaire	Study results
		Range of patient preference sensitive items	6 weeks—6 months	Weekly usage or at own pace, two exceptions: 4×/week and daily use		6/13 with HCP contact	Evenly divided between both options			All after treatment, one combined with during			9/13 with significant improvement
(van den Berg et al., 2015, 2012, 2013)	BREATH	Self‐management based on CBT components	4 months	1 h/week	Frequency: 0–45 logins (11 ± 7); Total duration: 0–2,324 min (337 ± 164); session duration: 24.7 ± 16.1 min; activity: 0–104 of 104 (50 ± 43) ingredients were opened	No	—	RCT	150 (133)	After treatment, 2–4 months	Secondary	CIS—fatigue	ES = 0.32, −4.144, 95% CI [−7.404, −0.884] (*p* < 0.05)*
(Kanera et al., 2017; Kanera, Bolman, et al., 2016; Kanera, Willems, et al., 2016; Willems et al., 2015; Willems, Bolman, et al., 2017; Willems, Lechner, et al., 2017; Willems, Mesters, et al., 2017)	Cancer aftercare guide [*Kanker Nazorg Wijzer*]	Tailoring and skills management, implemented using PST and CBT	6 months	Users can choose what modules to visit and what assignments to make	Participants used 2.22 ± 1.58 modules, average time between first and last login: 10.7 ± 6.8 weeks	No	—	RCT	462 (409)	After treatment, 4–56 weeks	Primary	CIS	B = −4.36, 95% CI [−8.03, −0.67] (*p* = 0.02), *f* ^2^ = 0.013, ES = 0.21 95% CI [0.02, 0.41]*
(Yun et al., 2012)	Health navigation	Individually tailored education programme based on transtheoretical model and social cognitive theory or CBT	12 weeks	At own pace	—	One of the components is named health professional monitoring, but no further explanation given	Unclear	RCT	273 (243)	After treatment, max. 24 months	Primary	BFI and FSS	BFI global: −0.66, 95% CI [−1.04, −0.27] (*p* = 0.001), ES = 0.29. FSS: −0.49, 95% CI [−0.78, −0.21] (*p* = 0.001), ES = 0.27*
(Owen et al., 2017)	Health‐space	Social networking intervention based on supportive‐expressive support group and coping skills training	12 weeks	Weekly new module topic with 90 min guided chat. Other than that, own pace	‐	Weekly chat is guided	Synchronous	Pilot RCT	347 (235)	Both during and after treatment	Secondary	POMS—Fatigue	ES = 1.19, 95% CI [0.01, 2.37] (*p* < 0.05)*
(Mendes‐Santos et al., 2019)	iNNOVBC	ACT‐influenced CBT intervention	10 weeks	60 min/module, 1 module/week	—	Weekly feedback from therapist, possibility for videoconferencing	Asynchronous	Protocol for RCT	Goal: 158	After treatment, 6 months‐10 years	Secondary	BFI and items of EORTC QLQC30 and QLQBR23	Ongoing
(Bray et al., 2017)	Insight	Cognitive rehabilitation with computerised neurocognitive learning	15 weeks	4 × 40 min/week	Average usage of 25.1 h (range: 0.2–55.8) of recommended 40 h. 27% completed programme	No	—	RCT	242 (192)	After treatment, 6–60 months	Secondary	FACIT‐F	2.44, 95%CI [0.25, 4.62] (*p* = 0.03)*
(Dozeman et al., 2017)	I‐sleep	CBT for insomnia	9 weeks	Six sessions, at least weekly	59% completed all sessions	Weekly feedback from coach	Asynchronous	Pre‐post testing	171 (100)	After treatment, 3 months‐5 years	Secondary	FSS	ES = 0.24, 95% CI [0.08, 0.39] (*p* < 0.01)*
(Kelleher et al., 2021)	mPCST‐community	PCST based on social cognitive theory	8 weeks	Daily use of mobile phone application, 4 × 50‐min video conference	—	Video conferencing session is guided and there is contact through the mobile phone application	Synchronous	Protocol for RCT	Goal: 180	After treatment, max. 3 years	Primary	PROMIS—Fatigue scale	Ongoing
(Abrahams et al., 2017, 2015)	On the road to recovery	CBT	6 months	8 modules, two‐weekly contact with therapist, no further timing indicated	Duration: 25 ± 4 weeks, e‐consultation consisted of on average 10 emails and 1 telephone/video consultation. 63–100% of the modules were indicated and opened	F2F session to start intervention, two‐weekly contact via email	Synchronous	RCT	132 (125)	After treatment, min. 3 months	Primary	CIS – Fatigue	ES = 1.0, 95% CI [0.6, 1.3] (*p* < 0.001)*
(Henry et al., 2018)	PROSPECT	CBT self‐management	8 weeks	Unrestricted access, at own pace	—	No	—	Pilot study	50 (45)	After treatment, min. 3 months	—	PROMIS 29 – Fatigue	ES = 0.84, −5.23 ± 8.0, (*p* < 0.001)*
(Corbett et al., 2016)	REFRESH	CBT	8–10 weeks	45–60 min/week	—	No	—	Protocol for RCT pilot	Goal: 80	After treatment, min. 3 months	Primary	PFS‐R	Ongoing
(Foster et al., 2015, 2016; Grimmett et al., 2013; Myall et al., 2015)	RESTORE	Increase self‐efficacy using verbal persuasion, goal setting, vicarious experience, psychosocial support, and CBT	6 weeks	30 min/week	71% logged on to session 1,2 and at least one third session. 60% did four sessions and 43% all sessions	No	—	Exploratory RCT	159 (118)	After treatment, max. 5 years	Secondary	BFI	0.353 95% CI [−0.293, 0.999] (*p* = 0.28)
(Ritterband et al., 2012)	SHUTi	CBT for insomnia	9 weeks	45–60 min/module, 6 modules, next opens week after completion of previous	Users logged in 15–61 times (38 ± 16), 86% completed all cores	No	—	RCT	28 (28)	After treatment	Secondary	MFSI‐SF	ES = 1.16 (*p* < 0.01)*
(Zachariae et al., 2018)					4.1 ± 2.5 cores were completed, 60% completed all cores				255 (203)			FACIT‐F	ES = 0.42, 95% CI [0.14, 0.70] *p* (<0.001)*

Abbreviations: ACT, acceptance and commitment therapy; BFI, Brief Fatigue Inventory; CBT, Cognitive Behavioural Therapy; CI, confidence interval; CIS, Checklist for Individual Strength; EORTC‐QLQ‐C30 and QLQ‐BR23, European Organization for Research and Treatment Cancer Quality of Life Questionnaire—general and breast cancer specfic; FACIT‐F, Functional Assessment of Chronic Illness Therapy—Fatigue; FSS, Fatigue Severity Scale; HCP contact, contact with healthcare professional; MFSI‐SF: Multidimensional Fatigue Symptom Inventory—Short Form; PCST, pain coping skills Training; PFS‐R, Piper Fatigue Scale—Revised; POMS, profile of mood states; PROMIS, Patient‐Reported Outcomes Measurement Information System; PST, problem solving therapy; RCT, randomised controlled trial.

* Significant improvement.

**TABLE 6:** Overview of interventions of another category than physical activity, mind–body or psychological and their aspects. In bolt at the top, the range of the patient preference sensitive attributes is summarised

Author/year	Name	Type of intervention	Duration	Intensity	Intensity of usage	HCP contact	(A)synchronous	Type of study	# participants [started (finished)]	Relation to treatment	Primary/secondary outcome	Questionnaire	Study results
		Range of patient preference sensitive items	6 months	Own pace—daily usage		2/2 with HCP contact	Synchronous and asynchronous			Both after treatment			1/2 with significant improvement
(Kapoor & Nambisan, 2020)	ACESO	Symptom and treatment‐related morbidity tracking combined with information and reminders related to the SCP.	6 months	Not described, usage at own pace	—	Nurse moderates the discussion forum	Asynchronous	Protocol for RCT pilot	Goal: 50	After treatment, max. 3 months	Secondary	BFI	Ongoing
(Cairo et al., 2020)	Vida	Health and wellness coaching	6 months	Daily motivation given by coach via the app	—	The wellness coach gives daily motivation and maintains contact with video/phone consultation	Synchronous	Non‐randomised 2‐group control design, pre‐post measures	147 (127)	After treatment	—	VAS—fatigue	Pre‐post intervention: delta = 1.2 ± 2.4 95% CI [0.7, 1.8] (*p* < 0.001)*

Abbreviations: BFI, Brief Fatigue Inventory; CI, confidence interval; HCP contact, contact with healthcare professional; RCT, randomised controlled trial; SCP, Survivorship Care Plan; VAS, visual analog scale.

* Significant improvement.

**TABLE 7:** Overview of interventions combining different categories of interventions and their aspects

Author/year	Name	Type of intervention	Duration	Intensity	Intensity of usage	HCP contact	(A)synchronous	Type of study	#participants [started (finished)]	Relation to treatment	Primary/secondary outcome	Questionnaire	Study results
		Range of patient preference sensitive items	8 weeks—6 months	Daily use–usage at own pace–once/twice per week		5/8 with HCP contact	About evenly divided between both options			2× during–5× after–2× both			All significant improvement
(Bruggeman‐Everts et al., 2015, 2017; Wolvers, 2017; Wolvers et al., 2015)	More fit after cancer [*fitter na Kanker*] – eMBCT	MBCT	9 weeks	30 min/day, 6 days/week, at most 4 h/week	38% were non‐adherent (adherence defined as following >70% of intervention)	Weekly feedback from therapist	Asynchronous	Pilot study	257 (159)	After treatment, min. 6 months	Primary	CIS	*t*(18) −13.27 (*p* < 0.001), ES = 1.45 (*)
								Three‐armed RCT	167 (139)	After treatment, min. 3 months			Chi^2(2) = 10.89 (*p* = 0.004), ES = 0.94 (*)
(Nápoles et al., 2019)	New Dawn [*Nuevo Amanecer*]	SCP programme with activity tracker, phone calls based on social cognitive theory	2 months	Daily step goal, within 2 months, 5 times a weekly phone call	Calls lasted 15 ± 3.4 min, 19/23 completed all calls, app is synchronised 4.4–5.7 times per week and checked 4.2–5.9 times per week	5 weekly phone calls by health coach	Synchronous	Single‐arm feasibility/mixed methods	23 (21)	After treatment, max. 1 year	Primary	PROMIS – Cancer – Fatigue scale with adjustments	ES = 0.4, *B* = −0.26 (*p* = 0.02) (*)
(Holtdirk et al., 2021, 2020)	Optimune	CBT extended with mindfulness‐based techniques and dietary and physical activity advice	3 months	30–45 min/module, 16 modules	Intervention was used for 25.7 ± 33.9 days	No	—	RCT	363 (306)	After treatment, min. 1 month, max 5 years	Secondary	BFI	ES = 0.23 CI = 0.02 to 0.44 (*)
(Smith et al., 2019)	Reimagine	Coping skills training, mind–body therapy, and CBT	18 weeks	At own pace, access any time	—	Online introductory meeting is guided	Synchronous	RCT	122 (86)	Both during and after treatment	—	FACIT‐F	ES = 0.46, *t*‐test = 2.2 (*p* = 0.034) (*)
(Grossert et al., 2016; Urech et al., 2018)	STREAM	CBT and MBSR	8 weeks	Daily use of exercises, one module per week	Median duration: 11.7 (IQR 9.1–18.6) weeks. 80% used at least 6/8 modules, 75% worked with all modules	Weekly feedback given via email	Asynchronous	RCT	129 (117)	During treatment	Primary	FACIT‐F	4.51 95% CI [1.81, 7.22] (*p* = 0.002) (*)
(Spahrkäs et al., 2020a, 2020b)	Untire	CBT and psychoeducation, MBSR, exercise instructions and positive psychology	12 weeks	At own pace, daily use is recommended, preferably at least once a week	Three equal groups, high users (33%): ≥ 9 days in total, medium users (33%): ≥ 3 days total, low users (33%): ≥ 1 day total	No	—	RCT	799 (335)	Both during and after treatment	Primary	FSI	Fatigue severity: F(3,1912) = −4.55 (*p* < 0.01), ES = 0.40. Fatigue interference: F(3,1912) = −4.10 (*p* < 0.01), ES = 0.35 (*)
(Zhou et al., 2020)	WeChat	Rehabilitation on physical, psychological, and social aspects, using the HBM framework	6 months	Daily rehabilitation information is provided	—	A group of a doctor, nurse, researchers, and postgraduate trainee provided daily information	Asynchronous	RCT	111 (103)	During treatment	Secondary	NRS	No group effect, there is a time effect: F = 3.52 (*p* = 0.02) (*)
(Lee et al., 2014)	WSEDI	Exercise and diet intervention, based on the TTM theory	12 weeks	Use regularly, at least twice per week	89% of the participants consistently participated in the programme	No	—	Pilot RCT	59 (57)	After treatment, max. 12 months	Secondary	BFI	*p* = 0.032 (*)

Abbreviations: BFI, Brief Fatgue Inventory; CI, confidence interval; CBT, cognitive behavioural therapy; CIS, Checklist for Individual Strength; FACIT‐F, Functional Assessment of Chronic Illness Therapy—Fatigue; FSI: Fatigue Symptom Inventory; HBM, health belief model; HCP contact, contact with healthcare professional; MBCT, Mindfulness‐based Cognitive Therapy MBSR, Mindfulness‐based Stress Reduction; NRS, Numeric Rating Scale; PROMIS, Patient‐Reported Outcomes Measurement Information System; RCT, randomised controlled trial; SCP, survivorship care plan; TTM, transtheoretical model.

* Significant improvement.

### 3.2 (Preference sensitive) attributes of interventions

For the second research question, ‘what (preference sensitive) attributes make up these interventions?’, the (preference sensitive) attributes as listed in Table 2 are compared below. First, the attributes that are preference sensitive are described, after which additional attributes are described that might be relevant to patients as well. These are the usage of and experiences with the intervention and the timing of the intervention relative to primary cancer treatment. Last, patient characteristics in relation to dropping out and being adherent are described. A preliminary version of the results was discussed in the consultation sessions.

#### 3.2.1 Duration

The duration of interventions varied both within and between categories. The categories physical activity, psychological and combination of interventions had the widest range of duration, from 6 weeks to 6 months. Mind–body interventions lasted shorter, varying between 4 and 12 weeks with one outlier of 20 weeks. The interventions in the ‘other’ category both had a duration of 6 months.

#### 3.2.2 Intensity

Most (3/5) physical activity interventions expected the participants to have three sessions per week (Delrieu et al., 2018; Falz et al., 2021; Galiano‐Castillo et al., 2013). The mind–body interventions all had daily practicing of exercises (Carlson et al., 2019; Kubo et al., 2018; Lengacher et al., 2018; Mikolasek et al., 2018; Price‐Blackshear et al., 2020; Subnis et al., 2020; Zernicke et al., 2013). In contrast, most psychological interventions (11/13), participants had weekly usage or at their own pace (Abrahams et al., 2015; Corbett et al., 2016; Dozeman et al., 2017; Grimmett et al., 2013; Henry et al., 2018; Mendes‐Santos et al., 2019; Owen et al., 2017; Ritterband et al., 2012; van den Berg et al., 2012; Willems et al., 2015; Yun et al., 2012). For the interventions consisting of a combination of categories, the expected intensity of use varied between usage at own pace (2/8) (Holtdirk et al., 2020; Smith et al., 2019), once or twice a week (2/8) (Lee et al., 2014; Spahrkäs et al., 2020a) and daily (4/8) (Grossert et al., 2016; Nápoles et al., 2019; Wolvers et al., 2015; Zhou et al., 2020). One of the interventions in the ‘other’ category had daily interaction with a coach (Cairo et al., 2020), whereas the other was used at own pace (Kapoor & Nambisan, 2020).

#### 3.2.3 Introduction training

Of the 35 interventions, 24 described to have some form of introduction to the interventions. Some of the physical activity interventions (3/5) have a face‐to‐face introduction (Delrieu, Anota, et al., 2020; Falz et al., 2021; Galiano‐Castillo et al., 2016). The mind–body interventions with introduction training (4/7) have either a face‐to‐face introduction (Kubo et al., 2018; Zernicke et al., 2016) or the introduction via an email or brochure (Lengacher et al., 2018; Subnis et al., 2020). Introductions in the psychological interventions (10/13) were mostly via an introductory page, module, or email, or a pop‐up at first log‐in (Abrahams et al., 2015; Corbett et al., 2016; Grimmett et al., 2013; Henry et al., 2018; Mendes‐Santos et al., 2019; Ritterband et al., 2012; van den Berg et al., 2012; Willems et al., 2015; Yun et al., 2012), although some were given (also) face‐to‐face (Abrahams et al., 2015; Kelleher et al., 2021; Mendes‐Santos et al., 2019; Ritterband et al., 2012; Yun et al., 2012). Both introductions in the other category (2/2) were face‐to‐face (Cairo et al., 2020; Kapoor & Nambisan, 2020). Introductions in the combination category (5/8) were both face‐to‐face introductions (Lee et al., 2014; Nápoles et al., 2019; Smith et al., 2019) and textual (module, brochure, email and pop‐up) (Holtdirk et al., 2020; Lee et al., 2014; Spahrkäs et al., 2020a).

#### 3.2.4 HCP contact

For all categories, there were interventions with HCP contact. For the physical activity interventions, 4/5 had HCP contact (Delrieu et al., 2018; Falz et al., 2021; Galiano‐Castillo et al., 2013; Wolvers et al., 2015), of which three had synchronous contact. In 2/7 mind–body interventions, professionals were involved synchronously (Carlson et al., 2019; Zernicke et al., 2013). Six of the 13 psychological interventions had professional involvement. Two had synchronous contact (Kelleher et al., 2021; Owen et al., 2017), two asynchronous contact (Dozeman et al., 2017; Mendes‐Santos et al., 2019), one intervention started with two face‐to‐face synchronous sessions, after which contact continued asynchronously via email (Abrahams et al., 2015) and one had ‘health professional monitoring’ without description on whether this is (a)synchronous (Yun et al., 2012). Both interventions in the ‘other’ category had professional involvement (Cairo et al., 2020; Kapoor & Nambisan, 2020), one synchronous and the other asynchronous, and in the combined interventions, 5/8 had professional involvement (Grossert et al., 2016; Nápoles et al., 2019; Smith et al., 2019; Wolvers et al., 2015; Zhou et al., 2020), of which three had asynchronous contact by giving daily or weekly written information.

The remaining 16 interventions without HCP contact can be followed anonymously. For some, it is necessary to have an email address or mobile phone number to create an account. Of note is that in a study setting, for some interventions, the research team was available to help with technical issues or called to stimulate adherence (Corbett et al., 2016; Henry et al., 2018; Lengacher et al., 2018; Puszkiewicz et al., 2016; Subnis et al., 2020; van den Berg et al., 2012; Zachariae et al., 2018).

#### 3.2.5 Peer support

Peer support was included in only seven interventions. One of the ‘combined’ interventions had an introductory group meeting (Smith et al., 2019), whereas in other interventions (psychological, Owen et al., 2017; Willems et al., 2015, and ‘other’, Kapoor & Nambisan, 2020) peer support was facilitated through an online discussion forum or chat sessions. Two mind–body interventions had weekly interactive sessions with peers (Carlson et al., 2019; Zernicke et al., 2016) and in one ‘combined’ intervention face‐to‐face peer contact was arranged (Zhou et al., 2020).

#### 3.2.6 Mode of content delivery

There were different aspects that together describe the content of interventions. Twenty‐two of the interventions were web‐based (Abrahams et al., 2015; Carlson et al., 2019; Corbett et al., 2016; Dozeman et al., 2017; Galiano‐Castillo et al., 2013; Grimmett et al., 2013; Grossert et al., 2016; Henry et al., 2018; Holtdirk et al., 2020; Kapoor & Nambisan, 2020; Lee et al., 2014; Mendes‐Santos et al., 2019; Owen et al., 2017; Price‐Blackshear et al., 2020; Ritterband et al., 2012; Smith et al., 2019; van den Berg et al., 2012; Willems et al., 2015; Wolvers et al., 2015; Yun et al., 2012; Zernicke et al., 2016; Zhou et al., 2020), ten were via an application on a phone or tablet (Cairo et al., 2020; Delrieu et al., 2018; Falz et al., 2021; Kelleher et al., 2021; Lengacher et al., 2018; Mikolasek et al., 2021; Nápoles et al., 2019; Puszkiewicz et al., 2016; Spahrkäs et al., 2020b; Subnis et al., 2020), two were used both via a website and an app (Kubo et al., 2018; Wolvers et al., 2015) and one intervention was delivered via a compact disc (Bray et al., 2017). Most physical activity interventions (4/5) had an app, whereas most psychological interventions (11/13) were web‐based.

Information was presented to users in various ways, namely, via video (*n* = 21), audio (*n* = 16), text (*n* = 26) or with images/graphics and visualisation (*n* = 11). Most psychological interventions used text and audio, whereas all mind–body interventions had audio tracks. There were 21 interventions that had assignments, assessments or exercises, that is, in physical activity, psychological or combined interventions. Some interventions had other options, like vignettes with information (*n* = 2, psychological interventions), quizzes (*n* = 3, physical activity and psychological interventions) or an activity tracker to support in reaching activity goals (*n* = 2, physical activity and combined interventions). Table S1 describes the modes of content delivery per intervention.

#### 3.2.7 Costs

Only six studies described the costs of their interventions. For two interventions, parts of the sessions or exercises in the app were free of charge with the possibility to buy more content (Puszkiewicz et al., 2016; Subnis et al., 2020). Two interventions described that participants of the study had the possibility to use the intervention free of charge, indicating that otherwise costs were involved (Smith et al., 2019; Spahrkäs et al., 2020a). The e‐mindfulness‐based cognitive therapy (eMBCT) intervention with weekly HCP feedback was covered by insurance (Bruggeman‐Everts et al., 2015) and the health and wellness coaching app with daily HCP contact had a subscription fee of $65 per month (Cairo et al., 2020).

#### 3.2.8 Study results (effectiveness)

In Tables 3, 4, 5, 6, 7, the results of the interventions on the various fatigue outcomes are shown, showing that 24 (69%) interventions had significant results. Four (11%) interventions did not find significant results and for eight (23%) interventions, a study was still ongoing. One intervention is counted twice as the feasibility trial had nonsignificant results, but the RCT is still ongoing (Delrieu, Anota, et al., 2020; Delrieu, Pialoux, et al., 2020).

In all categories, there was at least one intervention that had a significant effect on CRF. In physical activity interventions, 2/5 interventions found significant results (Bruggeman‐Everts et al., 2017; Galiano‐Castillo et al., 2017). For mind–body interventions, this was the case in 4/7 interventions (Lengacher et al., 2018; Mikolasek et al., 2021; Price‐Blackshear et al., 2020; Zernicke et al., 2014) and with regard to the psychological interventions, 9/13 found a significant effect on CRF (Abrahams et al., 2017; Bray et al., 2017; Dozeman et al., 2017; Henry et al., 2018; Owen et al., 2017; Ritterband et al., 2012; van den Berg et al., 2015; Willems, Bolman, et al., 2017; Yun et al., 2012; Zachariae et al., 2018). Of the two interventions of the ‘other’ category, one had a significant improvement (Cairo et al., 2020) and in the combined category, for all interventions, a significant improvement in CRF was found (Bruggeman‐Everts et al., 2017; Holtdirk et al., 2021; Lee et al., 2014; Nápoles et al., 2019; Smith et al., 2019; Spahrkäs et al., 2020b; Urech et al., 2018; Zhou et al., 2020).

### 3.3 Usage

Of the preference sensitive attributes described above, duration and intensity are both related to expected usage. However, it is also relevant to know whether patients used the intervention as proposed. For 21 interventions, a form of usage by participants was reported (see Table S1). This was the case in 3/5 exercise (Delrieu, Pialoux, et al., 2020; Galiano‐Castillo et al., 2016; Puszkiewicz et al., 2016), 5/7 mind–body (Kubo et al., 2018; Lengacher et al., 2018; Mikolasek et al., 2018; Price‐Blackshear et al., 2020; Zernicke et al., 2014), 7/13 psychological (Abrahams et al., 2017; Bray et al., 2017; Dozeman et al., 2017; Foster et al., 2016; Ritterband et al., 2012; van den Berg et al., 2013; Willems, Bolman, et al., 2017; Zachariae et al., 2018) and 6/8 combined interventions (Bruggeman‐Everts et al., 2015; Holtdirk et al., 2021; Lee et al., 2014; Nápoles et al., 2019; Spahrkäs et al., 2020b; Urech et al., 2018).

Nine interventions reported on the intensity of use (Bray et al., 2017; Kubo et al., 2018; Lengacher et al., 2018; Mikolasek et al., 2018; Nápoles et al., 2019; Puszkiewicz et al., 2016; Spahrkäs et al., 2020b; van den Berg et al., 2013; Zernicke et al., 2014) and duration of use was described in four interventions (Abrahams et al., 2017; Holtdirk et al., 2021; Urech et al., 2018; Willems, Bolman, et al., 2017). A last option to report usage was related to whether and how much participants completed the programme, which was done in 14 interventions (Abrahams et al., 2017; Bray et al., 2017; Bruggeman‐Everts et al., 2015; Dozeman et al., 2017; Foster et al., 2016; Galiano‐Castillo et al., 2016; Lee et al., 2014; Mikolasek et al., 2018; Price‐Blackshear et al., 2020; Ritterband et al., 2012; Urech et al., 2018; van den Berg et al., 2013; Willems, Bolman, et al., 2017; Zachariae et al., 2018; Zernicke et al., 2014). In some studies, completion of the programme was combined with information on duration or intensity.

### 3.4 Experiences with intervention

For 21 interventions, patients were asked for their experience with the intervention. Experiences were asked both qualitative and quantitative. Overall, patients were positive about the interventions, although sometimes technological issues were reported. In Table S1, experiences are described more extensively.

### 3.5 Cancer treatment

The timing of the intervention in relation to the primary treatment of cancer differs. Some interventions were delivered during primary treatment, whereas others were delivered after primary cancer treatment. For interventions after treatment, it differed whether these were in the first 5 years after treatment or in a later phase (Fosså et al., 2008; Mols et al., 2005; Thong et al., 2009). Division into these three periods is specified in Table S1.

Almost all interventions were for patients after primary treatment. Five interventions were delivered only during primary treatment; these were one physical activity intervention (Delrieu, Anota, et al., 2020), two mind–body interventions (Carlson et al., 2019; Kubo et al., 2018) and two interventions of the combined category (Grossert et al., 2016; Zhou et al., 2020). Four interventions were delivered to patients both during and after treatment; these were one mind–body intervention (Mikolasek et al., 2021), one psychological intervention (Owen et al., 2017) and two in the combined category (Smith et al., 2019; Spahrkäs et al., 2020a).

Eleven interventions after cancer treatment were delivered to patients both within and after the first 5 years since diagnosis, namely, 3/5 physical activity (Galiano‐Castillo et al., 2013; Puszkiewicz et al., 2016; Wolvers et al., 2015), 1/7 mind–body (Subnis et al., 2020), 5/13 psychological (Abrahams et al., 2015; Corbett et al., 2016; Henry et al., 2018; Mendes‐Santos et al., 2019; Ritterband et al., 2012), 1/2 other (Cairo et al., 2020) and 1/8 combined interventions (Bruggeman‐Everts et al., 2015). The other interventions were delivered only within the first 5 years after diagnosis, sometimes in a more specified period (see also Tables 3, 4, 5, 6, 7, Bray et al., 2017; Dozeman et al., 2017; Falz et al., 2021; Grimmett et al., 2013; Holtdirk et al., 2020; Kapoor & Nambisan, 2020; Kelleher et al., 2021; Lee et al., 2014; Lengacher et al., 2018; Nápoles et al., 2019; Price‐Blackshear et al., 2020; van den Berg et al., 2012; Willems et al., 2015; Yun et al., 2012; Zernicke et al., 2013).

### 3.6 Patient characteristics

Tables 8, 9, 10, 11 report information on reasons for drop‐out, characteristics of drop‐outs, characteristics of (non‐)adherent participants and other relations found between patient characteristics and outcome variables. It varied whether studies reported such information and what specific information was given. Therefore, no comparison was made between (categories of) interventions.

**TABLE 8:** Reasons reported for dropping out the various studies

(Contrast with) expectation of intervention/study	Did not meet expectation (Abrahams et al., 2017; Bruggeman‐Everts et al., 2015) App was annoying (Cairo et al., 2020) Too much work (Abrahams et al., 2017; Bruggeman‐Everts et al., 2015, 2017; Cairo et al., 2020; Price‐Blackshear et al., 2020) Busy (Galiano‐Castillo et al., 2016; Lee et al., 2014; Yun et al., 2012; Zernicke et al., 2014) Time constraints/lack of time (Smith et al., 2019; Urech et al., 2018) Dissatisfied with programme (Urech et al., 2018) Lack of confidence in usefulness (Bruggeman‐Everts et al., 2017) Preference for f2f (Bruggeman‐Everts et al., 2017) Difficulty integrating in daily life (Bruggeman‐Everts et al., 2015) Mindfulness did not suit (Bruggeman‐Everts et al., 2015)
Technical issues	Technical problems (Bray et al., 2017; Bruggeman‐Everts et al., 2017) Lack of computer skills (Urech et al., 2018; Zernicke et al., 2014) Poor usability of aspects of intervention (Bruggeman‐Everts et al., 2017) Difficulty with online communication (Bruggeman‐Everts et al., 2017) Poor internet use (Yun et al., 2012) Internet guidance not suitable (Bruggeman‐Everts et al., 2015)
Health issues	Unwell (Bray et al., 2017; Kubo et al., 2018; Price‐Blackshear et al., 2020; Smith et al., 2019; Zernicke et al., 2014) Passed away (Cairo et al., 2020; Delrieu, Pialoux, et al., 2020; Galiano‐Castillo et al., 2016; Urech et al., 2018; Willems, Bolman, et al., 2017) Recurrence/diagnosis of metastases (Abrahams et al., 2017; Cairo et al., 2020; Kubo et al., 2018; Lee et al., 2014; Yun et al., 2012) Had baby (Price‐Blackshear et al., 2020) Fatigue had reduced (Bruggeman‐Everts et al., 2015, 2017) Health reasons (Galiano‐Castillo et al., 2016; Urech et al., 2018) Co‐morbid illness got worse (Bruggeman‐Everts et al., 2015)b
Lost to follow‐up	Lost to follow‐up (Bray et al., 2017) Lost contact (Foster et al., 2016; Smith et al., 2019; Urech et al., 2018) Declined to answer questions (Cairo et al., 2020)
Other	Personal reasons (Galiano‐Castillo et al., 2016) Dissatisfied with allocation (Price‐Blackshear et al., 2020; Urech et al., 2018) Not following intervention as supposed (Kubo et al., 2018; Price‐Blackshear et al., 2020) Scheduling issues (Kubo et al., 2018; Zernicke et al., 2014) Moved for treatment (Zernicke et al., 2014) Individual counselling (Zernicke et al., 2014) Unwilling to further participate (Abrahams et al., 2017; Cairo et al., 2020; Urech et al., 2018) Not specified/other (Bray et al., 2017; Delrieu, Pialoux, et al., 2020; Foster et al., 2016; Urech et al., 2018; Willems, Bolman, et al., 2017; Yun et al., 2012)

**TABLE 9:** Characteristics reported of the drop‐outs in the various studies

Demographics	No difference in age (Henry et al., 2018) Being younger (Bray et al., 2017) Men (Bruggeman‐Everts et al., 2015; Willems, Bolman, et al., 2017) Lower educational level (Bruggeman‐Everts et al., 2015; Kubo et al., 2018) Lower income (Kubo et al., 2018) Higher social functioning (Willems, Bolman, et al., 2017) Less occupied with household activities (Bruggeman‐Everts et al., 2015) Shorter time since diagnosis (Kubo et al., 2018)
Health	Higher rates of antidepressant use (Bray et al., 2017) Suffering from fatigue shorter (Bruggeman‐Everts et al., 2015) Poorer quality of life (Bruggeman‐Everts et al., 2015) Less good prognosis (Bruggeman‐Everts et al., 2015) More comorbidities (Bruggeman‐Everts et al., 2015) Less often breast cancer (Bruggeman‐Everts et al., 2015) More often ‘other’ cancer (Bruggeman‐Everts et al., 2015) Less often already received help to manage fatigue (Spahrkäs et al., 2020b)
Other	No difference between any of the background/outcome variables (Bruggeman‐Everts et al., 2017; Zachariae et al., 2018) Being in intervention group (Willems, Bolman, et al., 2017; Zernicke et al., 2014)

**TABLE 10:** Characteristics and relations reported of (non‐)adherent participants in the various studies

Intervention usage	Higher baseline anxiety/depression leads to less training time (Bray et al., 2017) More module use was predicted to the indicatory red/orange ‘traffic lights’ (Kanera, Willems, et al., 2016) More module use was predicted by higher perceived relevance of modules (Kanera, Willems, et al., 2016) Less module use was related with having a partner (Kanera, Willems, et al., 2016)
Baseline/personality characteristics	Adherent users are female, non‐adherent users are male (Bruggeman‐Everts et al., 2015; Mikolasek et al., 2018) Adherent users have (Mikolasek et al., 2018) Higher openness to experience Higher depression on HADS Higher resistance to change Non‐adherent users have (Bruggeman‐Everts et al., 2015) More often depression at baseline Lower education Less often a paid job Less use of sleep medication No previous experience with mindfulness Non‐completers have/are (Dozeman et al., 2017) More often daytime fatigue More often on sick leave Baseline characteristics/levels (e.g. age, baseline symptom, motivation, expectation) did not predict adherence (Henry et al., 2018; Owen et al., 2017; van den Berg et al., 2013; Zachariae et al., 2018)

**TABLE 11:** Other relations reported between patient characteristics and outcome variables

Baseline characteristics/co‐morbidities	No difference in improvement of outcome by age (Henry et al., 2018) Younger (<56) participants had larger effect on fatigue (Spahrkäs et al., 2020b; Willems, Mesters, et al., 2017) Baseline levels of fatigue (Owen et al., 2017), demographic/clinical variables (Smith et al., 2019; Zernicke et al., 2016) and education and cancer status (Spahrkäs et al., 2020b) were no predictors of outcome on fatigue
Outcome variables	Fatigue was correlated with depression (Willems, Lechner, et al., 2017) and sitting time (Delrieu, Pialoux, et al., 2020) Change in self‐efficacy score was associated with fatigue symptoms (Smith et al., 2019) Clinically significant improvement is predicted by (Yun et al., 2012): Being moderately to severely fatigued Having sleep problems at baseline Having comorbidities at baseline More evident effect of intervention is seen with (Yun et al., 2012): Lower Brief Pain Inventory (BPI) severity score Higher sleep quality index I and II score
Adherence	Effect on fatigue was found when users used the module fatigue (Willems, Bolman, et al., 2017) Number of cores completed was not associated with improvement in fatigue (Zachariae et al., 2018) High/medium users had more reduction in fatigue than low/non users (Spahrkäs et al., 2020b)

Reasons for drop‐out (Table 8) were related to the expectation patients had of the intervention/study, technical and health issues, other reasons, or patients were lost to follow‐up. The characteristics of drop‐outs (Table 9), (non‐)adherent patients (Table 10) and other characteristics/relations (Table 11) were only described in 15 interventions (Bray et al., 2017; Bruggeman‐Everts et al., 2017; Delrieu, Pialoux, et al., 2020; Dozeman et al., 2017; Henry et al., 2018; Kubo et al., 2018; Mikolasek et al., 2018; Owen et al., 2017; Smith et al., 2019; Spahrkäs et al., 2020b; van den Berg et al., 2013; Willems, Bolman, et al., 2017; Yun et al., 2012; Zachariae et al., 2018; Zernicke et al., 2014). Characteristics were related to demographics and health status at baseline, and relations were found between CRF and adherence or CRF and other outcome variables. Not all interventions found specific characteristics for drop‐outs or adherent patients, some studies reported on a comparison in which no differences were found (Bruggeman‐Everts et al., 2017; Henry et al., 2018; Owen et al., 2017; Smith et al., 2019; van den Berg et al., 2013; Zachariae et al., 2018), and results differed per study. For example, in one study on a psychological intervention (Insight, Henry et al., 2018), there was no difference in age when comparing drop‐outs to the participants, whereas another study, also on a psychological intervention (PROSPECT, Bray et al., 2017), revealed that drop‐outs were younger.

## 4 DISCUSSION

For the first aim of our scoping review, we found 35 eHealth interventions for breast cancer patients with CRF that could be divided into the categories physical activity, mind–body, psychological, ‘other’ or a combination of categories. The second aim was to develop an overview of (preference sensitive) attributes that make up these interventions, showing there is much variation in attributes, both between and within the different categories.

This variation in attributes presents opportunity to personalise treatment advice towards preferences of an individual patient. Nevertheless, in preference studies, results are usually summarised for the participant group as a whole. For example, participants in the discrete choice experiment (DCE) of Phillips et al. (2021) preferred online interventions with lower costs, proven effectiveness and face‐to‐face contact with professionals and peers. Fortunately, next to these overall results, preference studies do perform subgroup analyses, showing that overall preferences do not hold for subgroups (Phillips et al., 2021; Sullivan & Hansen, 2017). This supports our idea that the variation in attributes is relevant and necessary to personalise treatment advice to preferences of patients.

In our review, we were specifically interested in the (preference sensitive) attributes of the interventions, without linking these to effectiveness. In the meta‐analysis of Mustian et al. (2017), additional analyses were done to investigate the effectiveness of individual variables, or attributes, of interventions. They found varying effect sizes for amongst others timing of intervention related to cancer treatment, HCP and/or peer contact and mode of content delivery (Mustian et al., 2017). Such additional subgroup analyses per attribute are useful and relevant in relation to personalised treatment recommendations. A next step would be to investigate this further, by looking in more details into ‘what works for whom?’ as this might be related to the preference sensitive attributes.

One interesting finding in relation to the review of Mustian et al. (2017) is that even though we did not aim to find effect sizes per category or attribute, we found that in the combination category, all interventions had a significant effect on CRF. Additionally, in psychological interventions, this was the case for 9/13 interventions, with the other four still ongoing. This is in line with their results after primary treatment, where psychological interventions with or without exercise have a larger effect size than exercise alone (Mustian et al., 2017).

Previous reviews on (eHealth) interventions also reported their results in Tables 3, 4, 5, 6, 7 with information on a number of intervention aspects, like content, mode of content delivery, duration, intensity and treatment status of patients (Corbett et al., 2019; Seiler et al., 2017; Vannorsdall et al., 2020; Xie et al., 2020). These reviews mostly continue with a meta‐analysis, whereas our goal was to use these tables to develop an overview of (preference sensitive) attributes. Like us, these reviews found variation, or heterogeneity, in the included interventions. Reasonably, this was seen as an disadvantage, as heterogeneity makes it more difficult or even impossible to pool results to find an overall effectiveness (Corbett et al., 2019; Seiler et al., 2017; Vannorsdall et al., 2020; Xie et al., 2020). In contrast, we see this variation as an opportunity for patients to select an intervention that matches their preferences and characteristics best. Additionally, next to using the variation to determine ‘what fits whom best’, again, it is also interesting to investigate ‘what works best for whom.’

### 4.1 (Preference sensitive) attributes of interventions

As previous studies have not extensively discussed the variation in attributes between interventions, below, we discuss some of the distinctions found in this scoping review.

The intensity of an intervention seemed to be related to the category of intervention: mind–body interventions had daily expectations, psychological interventions weekly usage and exercise interventions were in between with three sessions per week. This can be extended to the interventions that combine categories, as these had varying intensities. It might be that the intensity of these interventions is related to either of the three options, depending on the category that prevails within the intervention.

Interventions without HCP contact were said to be anonymous. In some, as described in the results, there is still contact with the research team possible. However, this contact is non‐existent in a non‐study setting, and therefore, these interventions were noted down as anonymous for patients.

Costs were only reported in six interventions. During a study, the intervention is still in a developing/testing phase, so the costs might still be unknown. However, costs are an important attribute to patients (Phillips et al., 2021) and, in line with inquiring experiences of users, costs might influence these results.

The mode of content delivery varied between the interventions included in this review. In the DCE of Phillips et al. (2021), only the main mode of content delivery was included, but in our overview, all options are included. As patients might like combinations as well, all modes of content delivery are relevant to report, instead of only the dominant one.

Even though usage is not reported in all studies, it is relevant to know if patients practiced with the intervention as supposed. On the one hand, this information specifically shows how well the expected usage of an intervention fitted patients and thus how well the intervention fitted the patients. On the other hand, if the intervention was effective while used less, it can also indicate that adjustments to the expected usage can be made. This then might result in a broader range of duration and intensity for this intervention and thus more patients with whom the preferences align.

The timing of the intervention relative to cancer treatment is based on the inclusion criteria of the interventions. However, this does not mean that in future prescription of these interventions, this is the only period in which patients can follow the intervention. It is the period for which the intervention is evaluated and thus the results are valid, but it is still possible to suggest the intervention to patients outside this range in case the intervention further fits the patients' preferences and characteristics.

### 4.2 Patient characteristics

The importance of asking patients' preferences and linking these to a suitable intervention is also shown by the reasons for drop‐out (Table 8). Preferences towards duration, intensity and HCP contact are relevant as well as technical issues patients might face.

Next to preferences, patient characteristics might be of importance to determine what intervention works best for which individual patient; however, this is not investigated sufficiently. In 15 interventions, characteristics were analysed and only one of those explicitly stated in their research question they were going to do this to determine the beneficial individual effect (Dozeman et al., 2017). Some studies focussed on just one characteristic, for example age, instead of subgroup of patients in which differences can occur. Therefore, in future intervention studies, it is important to include an analysis on intervention effectiveness for subgroups of patients to help and identify patients for whom the intervention worked best.

### 4.3 Strength and limitations

The two consultation sessions organised with experts are a strength of this study. Using preliminary results as input, the discussion during these sessions gave new insights in important attributes by including the perspective of experts on CRF and the user perspective.

The quality of the individual studies was not assessed. This step is not necessary to reach the goal of developing an overview of interventions and their attributes, and in the PRISMA‐ScR checklist, the critical appraisal of individual sources of evidence is optional (Tricco et al., 2018).

In this review, we included 35 eHealth interventions for breast cancer patients experiencing fatigue. This patient group was selected because the number of survivors is rising (Netherlands Cancer Registry, 2021; World Health Organization, 2021) and patients experience barriers for face‐to‐face interventions (Stubblefield, 2017). It could be that we missed studies from the way our search string was set up, for example if fatigue were a secondary outcome and not mentioned in the title/abstract/keywords. Also, interventions could be missed because of the databases searched. Some interventions are used without being tested in research setting; however, these cannot be identified in a systematic way as we did leading up to the same level of proof. Next to that, there are more interventions for patients with (cancer‐related) fatigue that might not have been studied with breast cancer patients but can still be useful for them. As an example, the CBT for insomnia intervention included in this review, SHUTi (Ritterband et al., 2012; Zachariae et al., 2018), was tested (although not always on fatigue) in different participant groups (Christensen et al., 2016; Hagatun et al., 2018).

Even though it is possible we missed some eHealth interventions for breast cancer patients with CRF, and more interventions are being developed over time, our overview shows there is a lot of variation within attributes. For clinical implementation, when the overview is used to help patients and healthcare professionals selecting a fitting intervention, adding extra interventions will extend the intervention options, but most likely not the attribute ranges.

### 4.4 Future research

Future research could focus on the implementation of our overview in a healthcare setting to support each individual patient with an intervention for CRF that fits them best. For this, a number of next steps need to be taken. First, more research needs to be done into preferences breast cancer patients have in relation to the attributes found in this review. Next, a method has to be developed to link the preferences of an individual to interventions included in the overview of this scoping review. This leads to personalised treatment recommendations for the patient. With this, it is important that the recommendation is transparent in terms of why a certain intervention fits a patient. This will help both the healthcare professional as well as the patient.

## 5 CONCLUSION

In this scoping review, we created an overview of existing eHealth interventions for breast cancer patients with CRF. Interventions were divided into the categories physical activity, mind–body, psychological, ‘other’ or a combination of categories. This overview outlines that there is variation in preference sensitive attributes related to time investment, introduction training, HCP contact, peer support, mode of content delivery, costs and effectiveness of interventions. Future research could show how this overview creates possibilities for patients to follow an intervention that better aligns with their preferences. This will, ideally, increase adherence, effectiveness and satisfaction, decrease CRF and, with that, improve quality of life of patients after breast cancer.

## CONFLICT OF INTEREST

The authors have no conflict of interest to declare.

## Supporting information


**Table S1.** An Excel file

## Data Availability

All results are available in Table S1.
